# Proprotein Convertase Subtilisin Kexin Type 9 Inhibitors Reduce Platelet Activation Modulating ox-LDL Pathways

**DOI:** 10.3390/ijms22137193

**Published:** 2021-07-03

**Authors:** Vittoria Cammisotto, Francesco Baratta, Valentina Castellani, Simona Bartimoccia, Cristina Nocella, Laura D’Erasmo, Nicholas Cocomello, Cristina Barale, Roberto Scicali, Antonino Di Pino, Salvatore Piro, Maria Del Ben, Marcello Arca, Isabella Russo, Francesco Purrello, Roberto Carnevale, Francesco Violi, Daniele Pastori, Pasquale Pignatelli

**Affiliations:** 1 Department of General Surgery and Surgical Speciality “Paride Stefanini”, Sapienza University of Rome, 00161 Rome, Italy; vittoria.cammisotto@uniroma1.it; 2 I Clinica Medica, Department of Clinical Internal, Anaesthesiological and Cardiovascular Sciences, Sapienza University of Rome, 00161 Rome, Italy; francesco.baratta@uniroma1.it (F.B.); valentina.castellani@uniroma1.it (V.C.); cristina.nocella@uniroma1.it (C.N.); nicholas.cocomello@uniroma1.it (N.C.); maria.delben@uniroma1.it (M.D.B.); francesco.violi@uniroma1.it (F.V.); daniele.pastori@uniroma1.it (D.P.); 3 Istituto Pasteur Italia-Fondazione Cenci Bolognetti and Department of Medical-Surgical Sciences and Biotechnologies, Sapienza University of Rome, 04100 Latina, Italy; simona.bartimoccia@uniroma1.it,; 4 Department of Translational and Precision Medicine, Sapienza University of Rome, 00161 Rome, Italy; laura.derasmo@uniroma1.it (L.D.); marcello.arca@uniroma1.it (M.A.); 5 Department of Clinical and Biological Sciences, University of Turin, 10154 Turin, Italy; cristina.barale@unito.it (C.B.); isabella.russo@unito.it (I.R.); 6 Department of Clinical and Experimental Medicine, Internal Medicine, University of Catania, 95122 Catania, Italy; robertoscicali@gmail.com (R.S.); antonino.dipino@unict.it (A.D.P.); piro@unict.it (S.P.); francesco.purrello@unict.it (F.P.); 7 Department of Medical-Surgical Sciences and Biotechnologies, Sapienza University of Rome, 04100 Latina, Italy; roberto.carnevale@uniroma1.it; 8 Mediterranea Cardiocentro, 80133 Napoli, Italy

**Keywords:** platelets, ox-LDL, PCSK9, familial hypercholesterolemia, NOX2

## Abstract

**Background:** Proprotein convertase subtilisin kexin type 9 inhibitors (PCSK9i) lower LDL-cholesterol and slow atherosclerosis preventing cardiovascular events. While it is known that circulating PCSK9 enhances platelet activation (PA) and that PCSK9i reduce it, the underlying mechanism is not still clarified. **Methods:** In a multicenter before–after study in 80 heterozygous familial hypercholesterolemia (HeFH) patients on treatment with maximum tolerated statin dose ± ezetimibe, PA, soluble-NOX2-derived peptide (sNOX2-dp), and oxidized-LDL (ox-LDL) were measured before and after six months of PCSK9i treatment. In vitro study investigates the effects of plasma from HeFH patients before and after PCK9i on PA in washed platelets (wPLTs) from healthy subjects. **Results:** Compared to baseline, PCSK9i reduced the serum levels of LDL-c, ox-LDL, Thromboxane (Tx) B2, sNOX2-dp, and PCSK9 (*p* < 0.001). The decrease of TxB2 correlates with that of ox-LDL, while ox-LDL reduction correlated with PCSK9 and sNOX2-dp delta. In vitro study demonstrated that wPLTs resuspended in plasma from HeFH after PCSK9i treatment induced lower PA and sNOX2-dp release than those obtained using plasma before PCSK9i treatment. This reduction was vanished by adding ox-LDL. ox-LDL-induced PA was blunted by CD36, LOX1, and NOX2 inhibition. **Conclusions:** PCSK9i treatment reduces PA modulating NOX2 activity and in turn ox-LDL formation in HeFH patients.

## 1. Introduction

Proprotein convertase subtilisin kexin type 9 (PCSK9) plays a key role in lipid metabolism and atherothrombotic process binding low-density lipoprotein cholesterol receptors (LDLRs) and addressing them to lysosomes where they are degraded [1,2]. PCSK9 inhibitors (PCSK9i) are monoclonal antibodies binding circulating PCSK9 and preventing PCSK9 binding to the LDLRs. In this way, PCSK9i increase LDLRs expression on cells membrane and LDL-cholesterol (LDL-c) scavenge from circulation [3,4].

In several clinical studies, the PCSK9i, alirocumab, and evolocumab, have been shown to improve atherosclerosis [5,6] and prevent cardiovascular events [7,8]. While the lipid-lowering effect of PCSK9 inhibitors is well established, pleiotropic effect of PCSK9i on cellular mechanisms involved in atherogenesis and plaques complication is still debated [9,10,11,12]

A recent metanalysis evaluating a large cohort of patients (*n* = 28,319), showed that high circulating PCSK9 concentration significantly associated with increased risk of major adverse cardiovascular events (MACEs) [13].

Several studies demonstrated that PCSK9 circulating level directly enhances platelet activation (PA) and in vivo thrombosis suggesting a possible role of PA in inducing MACE in subjects with elevated PCSK9 levels [14,15]. Recently, Barale et al. [16] showed that up to 12 months treatment with PCSK9i impacts on platelet function in heterozygous familial hypercholesterolemia (HeFH). FH is a particularly attractive model as the genetic mutations result in persistent lifelong extremely raised LDL-c levels, premature coronary artery disease, and systemic atherosclerosis [17]. LDL-c play a key role in the thrombotic process. Indeed, LDL modification by phospholipid oxidation during the inflammatory and oxidative processes of plaque formation results in the generation of the prothrombotic oxidized-LDL (ox-LDL) [18] [19]. Dyslipidaemia induces the generation of ox-LDL, which in turn, facilitate platelet activation by binding scavenger receptors on platelet’s surface including LOX-1 and CD36 [18]. Once activated, platelets can oxidize LDLs, generating a positive feedback of platelet activation through the activation of NOX2 [18]. This enzymatic system seems to play a key role in PA as PCSK9 per se can induce NOX2 activation and once produced, ox-LDL also amplify PA by inducing NOX2 activation [19]. This intricated process can be monitored in vivo by dosing sNOX2-dp, a soluble peptide released upon NOX2 activation [20], and TXB_2_ a marker of PA induced by arachidonic acid pathway [21]. Furthermore, these markers were reported to be correlated with atherosclerosis and cardiovascular disease in other settings [14,22,23,24,25]. However, NOX2 activity modulation after PCSK9i treatment was never investigated and, while the inhibition of PA in patients treated with PCSK9i was previously demonstrated by Barale et al., whether this depends on free circulating PCSK9 reduction or on lowering oxLDL is still to be defined [14,15,16,26].

For this purpose, we conducted a multicenter before–after study in HeFH patients to evaluate if six months of PCSK9i treatment could inhibit PA by modulating ox-LDL production and ox-LDL pathway.

## 2. Results

### 2.1. Patients’ Characteristics

The median age of patients treated with PCSK9i was 57.7 ± 10.8 years and 36 out of 80 (45%) were female. The median BMI was 26.5 ± 4.2 kg/m^2^. Prior cardiovascular events were recorded in 51.5% of patients, 41 out of 41 had coronary heart disease and 2 had peripheral artery diseases in addition. Antiplatelet drugs were used by 42 out of 80 (52.5%) patients and the same number of smokers was recorded. As per inclusion criteria, none of the patients had diabetes and all the patients were on statin treatment (Table 1).

### 2.2. Effect of PCSK9i Treatment

**Figure 1:**
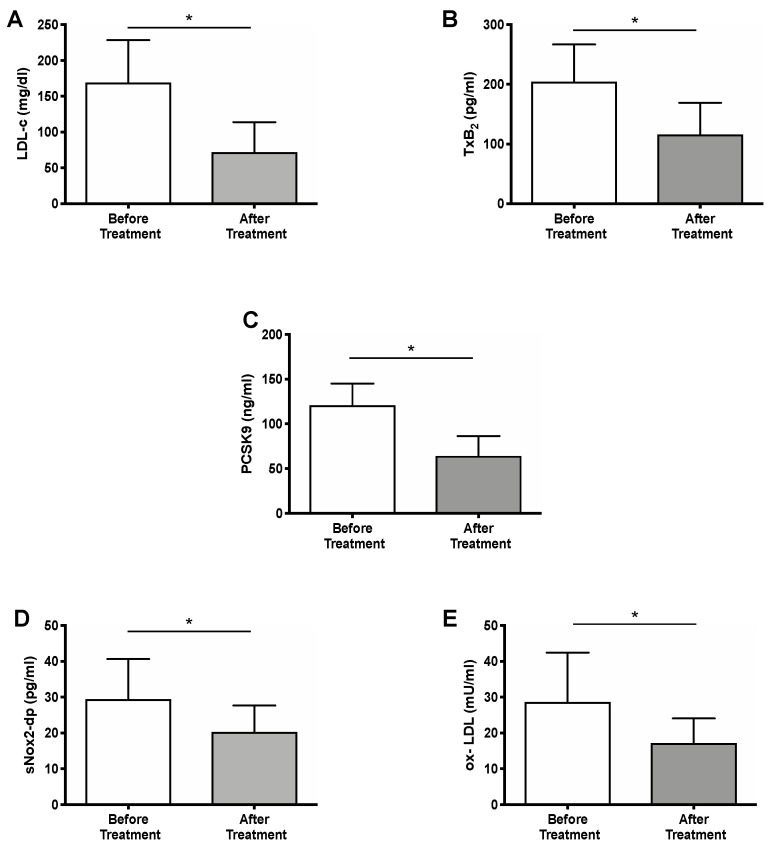
Ex vivo markers changes after PCSK9i treatment in HeFH patients. Plasma levels of LDL-c (**A**), TxB_2_ (**B**), PCSK9 (**C**), sNOX2-dp (**D**), and ox-LDL (**E**) production in 80 HeFH patients. (Data are represented as median and SD; * *p* < 0.0001). Abbreviation: PCSK9 inhibitors (PCSK9i); LDL-cholesterol (LDL-c); Thromboxane B_2_ (TxB_2_); soluble NOX2-derived peptide (sNOX2-dp), oxidized-LDL (ox-LDL), heterozygous familial hypercholesterolemia (HeFH).

After 6 months treatment with PCSK9i, LDL-c changed from 169.2 ± 59.4 to 71.9 ± 42.0 mg/dl, *p* < 0.001 (Figure 1, panel A). PA was significantly inhibited. Thus, TxB_2_ lowered from 204.3 ± 62.6 to 116.2 ± 52.9 pg/mL, *p* < 0.001, (Figure 1, panel B). There was also a reduction in circulating PCSK9 levels, changing from 121.0 ±24.1 to 64.3 ± 22.2 ng/mL (Figure 1, panel C). Concerning oxidative stress, we found that sNOX2-dp decreased from 29.5 ± 11.2 to 20.3 ± 7.4 pg/mL, *p* < 0.001 (Figure 1, panel D) and Ox-LDL from 28.6 ± 13.8 to 17.1 ±6.9 mU/mL, *p* < 0.001 (Figure 1, panel E).

A multivariate linear regression analysis showed that TxB_2_ reduction positively correlated with LDL-c (Beta:0.316; *p* = 0.007) and ox-LDL (Beta:0.233; *p* = 0.044). No correlations were found with PCSK9 and sNOX2-dp. Furthermore, ox-LDL reduction positively correlated with PCSK9 (Beta:0.277; *p* = 0.016) and sNOX2-dp variation (Beta:0.231; *p* = 0.045) (Table 2).

### 2.3. In Vitro Study

**Figure 2:**
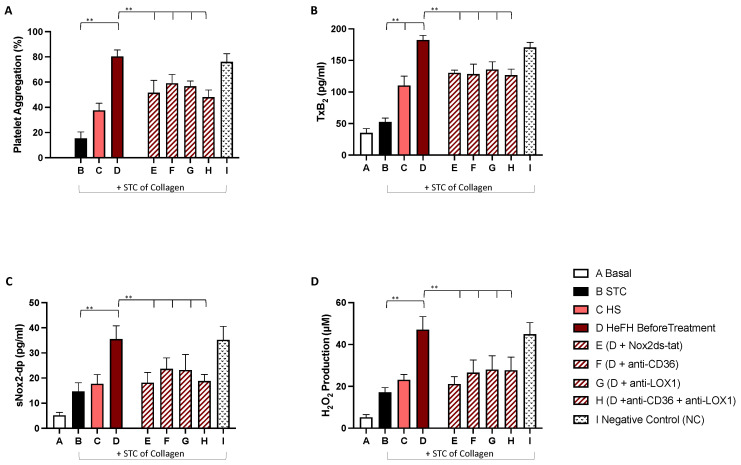
Platelet aggregation (**A**), TxB_2_ formation (**B**), sNOX2-dp release (**C**), and H_2_O_2_ production (**D**) evaluated in washed platelets from HS (*n* = 5) resuspended in plasma derived from HS or HeFH patients before treatment with PCSK9i and stimulated with STC of collagen (0.25 µg/mL) in presence or less of Nox2ds-tat or anti-CD36 or anti-LOX1 or NC. ** *p* < 0.001). Abbreviation: PCSK9 inhibitors (PCSK9i); Thromboxane B2 (TxB2); soluble NOX2-derived peptide (sNOX2-dp), hydrogen peroxide (H_2_O_2_); oxidized-LDL (ox-LDL), heterozygous familial hypercholesterolemia (HeFH), subthreshold concentration (STC), negative control (NC).

**Figure 3:**
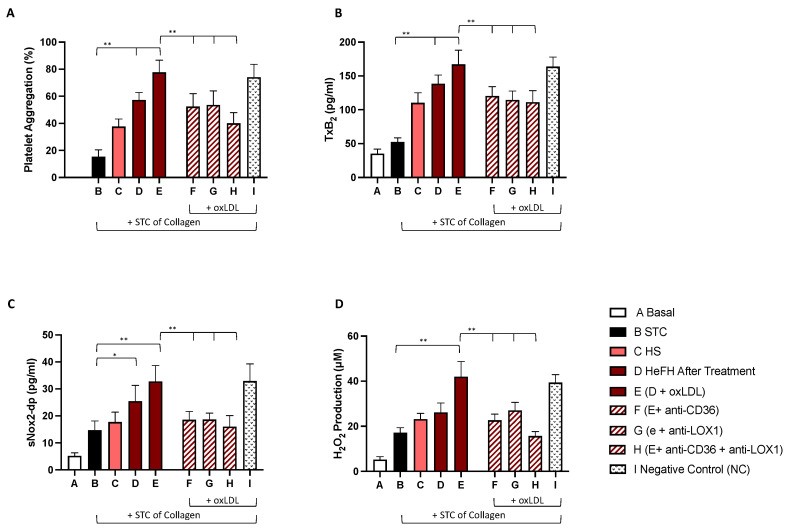
Platelet aggregation (**A**), TxB_2_ formation (**B**), sNOX2-dp release (**C**), and H_2_O_2_ production (**D**) evaluated in washed platelets from HS (*n* = 5) resuspended in plasma derived from HS or HeFH patients after treatment with PCSK9i and stimulated with STC of collagen (0.25 µg/mL) in presence or less of exogenous ox-LDL (25 mU/mL), Nox2ds-tat, anti-CD36, anti-LOX1, or NC (* *p* < 0.05; ** *p* < 0.001). Abbreviation: PCSK9 inhibitors (PCSK9i); Thromboxane B2 (TxB2); soluble NOX2-derived peptide (sNOX2-dp), hydrogen peroxide (H_2_O_2_); oxidized-LDL (ox-LDL), heterozygous familial hypercholesterolemia (HeFH), subthreshold concentration (STC), negative control (NC).

The results showed that plasma from HeFH patients enhanced PA and oxidative stress, as assessed by platelet aggregation, TxB2, sNOX2-dp, and H2O2, compared to plasma from HS (Figure 2 and Figure 3). Of note, PA and oxidative stress parameters were higher in wPLTs mixed with plasma taken from HeFH patients before treatment then wPLTs resuspended in plasma taken from HeFH patients after treatment. (Figure 2 and Figure 3, column D, panels A–D).

ox-LDL was 15.3 mU/mL and 26.4 mU/mL in plasma taken from HS and HeFH, respectively. Furthermore, to prove the role of NOX2, wPLTs were pretreated with NOX2ds-tat, a selective inhibitor of NOX2 activation, before being resuspended in plasma from HeFH patients before treatment. TxB2, sNOX2-dp, and H_2_O_2_ production were significantly reduced (Figure 2, columns E, Panels A–D). Similarly, we observed the same results incubating wPLTS mixed with plasma taken from HeFH patients before treatment and incubated with CD36 inhibitor. This evidence supports the hypothesis that NOX2 activation was due to CD36 pathway, which we previously demonstrated to be activated by PCSK9 [14] (Figure 2, columns F, panels A–D). CD36 is activated also by oxLDL. To prove a role of ox-LDL in the process, we pre-incubated wPLTS also with LOX1, observing the same results again. (Figure 2, columns G-H, panels A–D)

After PCSK9i treatment, the enhancement of PA by plasma from HeFH was no longer detected (Figure 3, columns C vs. D, Panels A). To assess the role ox-LDL, we added exogenous ox-LDL to plasma from patients after treatment. Platelet aggregation, TxB2, sNOX-dp levels, and H_2_O_2_ production were restored (Figure 3, columns E, Panels A–D). As further confirmation, pre-incubation with anti-CD36 and LOX1 reduced all marker levels (Figure 3, columns F-G, Panels A–D). Of note, the incubation with both inhibitors induced a further decrease of PA and oxidative stress markers. (Figure 3, columns H, Panels A–D).

## 3. Discussion

This study demonstrates for the first time that six months of PCSK9i treatment reduces platelet activation lowering circulating levels of ox-LDL and NOX2 activity and in patients with HeFH.

PCSK9i represent an important new tool in the management of hypercholesterolemia and cardiovascular prevention. Beyond the effect on LDL reduction, ranging from 50–60% [4,27] when used on top to statin ± ezetimibe therapy, PCSK9i achieved a reduction of about 15% of the risk to develop a cardiovascular event in secondary prevention [7,8]. This may suggest ancillary positive effects of PCSK9i on the thrombotic cascade involved in acute coronary events.

Evidence of interplay between PCSK9 and platelet activation were found in patients affected by atrial fibrillation (AF) [28] and recently confirmed by Barale et al. [16]. Indeed, they found in a small population of HeFH a correlation between circulating levels of platelet activation markers and PCSK9. Noteworthy, Barale and colleagues demonstrated that 6 months of PCSK9i administration reduced platelet activation. Nevertheless, they did not investigate the underling mechanism.

Here, we confirm, in a multicenter before–after study conducted on a larger population of HeFH patients, that PCSK9i reduces platelet activation and that this reduction correlated with oxLDL decrease. Moreover, oxLDL changes were significantly correlated to sNOX2-dp. As PCSK9 is able per se to activate NOX2, the concomitant decrease of both oxLDL and sNOX2-dp suggests that inhibiting circulating levels of free-PCSK9 could result in reduced PA via NOX2 activity modulation and ox-LDL formation. To support our hypothesis, we performed an in vitro study using platelets from HS mixed with plasma from HeFH before and after PCSK9i treatment. Platelet activation, in presence of post treatment plasma results in lower platelet aggregation and TxB_2_ production. At the same time, NOX2 activity was downregulated as shown by decreased sNOX2-dp release and formation of H_2_O_2_, a stable marker of NOX activity [20].

LDL oxidation plays a critical role in PA [18] by binding specific receptors on platelet surface, namely, CD36 and LOX1 [18,19]. Importantly, hyperlipidemic mice and humans with high LDL cholesterol have detectable levels of circulating oxidized lipids [18]. Although circulating levels of ox-LDL are not well defined, studies mixing “normal” platelets with plasma isolated from hyperlipidemic humans or mice (which contain detectable oxidized lipids) show platelet activation in a CD36 and LOX1-dependent manner. [18,19]. In our in vitro model, we confirm that ox-LDL both native from HeFH patients at baseline or added to samples post PCSK9i, were able to increase PA, a phenomenon that was counteracted by pretreating the samples with the specific ox-LDL receptor CD36 and LOX1 inhibitors. Of note, we obtain a similar inhibition by blocking the downstream pathways with a NOX2 inhibitor confirming that once bent to cell surface, ox-LDL need a pathway involving NOX2 activation in order to amplify PA.

We consider a limitation of our study that the reduction of ox-LDL concentration after PCSK9i treatment could be at least in part dependent on its ability to lower native LDL. However, at the multivariate linear regression analyses the delta reduction of ox-LDL did not significantly correlate to the delta reduction of native LDL, but only with that of sNOX2-dp and TxB_2_. Based on this, while recognizing the role of LDL reduction in reducing oxLDL formation and subsequent PA, the free-PCSK9 reduction after PCSK9i treatment might further decrease oxLDL formation, also stopping the known mechanism of PCSK9-dependent NOX2 activation [14]. Another possible limitation is the small sample size as well as the duration of the study to establish if this evidence might be persistent during a longer PCKS9i treatment. We found a decrease of PCSK9 circulating levels after treatment, differently from what was observed in other studies [29,30,31]. In fact, in three different prior studies, using an ELISA kit dosing both free PCSK9 and PCSK9-PCSK9i complex, an increase in PCSK9 levels was found after treatment. The evidence found in our study be due to the specify of our assay. probably, the assay we used measures the free-bound form of PCSK9. In fact, in a pooled analysis of clinical trials conducted on 3000 patients treated with evolocumab, using an assay built to detect free-PCSK9, authors found that PCSK9 decreased after treatment [32].

## 4. Materials and Methods

We conducted a prospective, multicenter before-after study in 80 non-diabetic HeFH patients treated with maximum tolerated statin dose ± ezetimibe who undergo treatment with PCSK9i. HeFH diagnosis was performed according to Dutch Lipid Clinic Network (DLCN) score [33]. PCKS9i were prescribed according to the Italian regulations [34] in patients with DLCN greater than 8 or with a molecular confirmation of HeFH. All patients have been included in the LIPIGEN study and had confirmed molecular diagnosis of HeFH [35,36]. Diabetic patients are known to be affected by impaired PA [37]. For this reason, diabetic patients were excluded to reduce confounding factors in PA evaluation.

Chronic infection, systemic autoimmune disease, diabetes, history of alcohol or drug abuse, cancer or liver insufficiency were criteria of exclusion for enrolment in the study.

Anamnestic, demographic, anthropometric, and routinary laboratory data were collected at baseline (defined as the visit when PCKS9i were prescribed), and after 6 months of PCSK9i treatment. A blood sample was collected at baseline and after 6 months of therapy.

Samples were collected by antecubital vein after a 12 h overnight fast. Plasma was stored at −70 °C until the analysis. Experimental marker analyses was performed centrally in Sapienza–University of Rome laboratory. For the in vitro study, blood samples obtained from HeFH patients were taken into tubes with 3.8% sodium citrate and centrifuged at 300 g for 10 min to obtain supernatant, then immediately stored at −80 °C.

All subjects gave written informed consent to be included in the study. The study protocol was approved by the local ethical board of Sapienza University of Rome (ref n° 3939) and was conducted according to the principles of the Declaration of Helsinki.

**Plasma and platelets sNOX2-dp production**

Plasma and platelets NOX2 activation was measured as a soluble NOX2-derived peptide (sNOX2-dp) with an ELISA method as previously reported [38]. Briefly, the peptide is recognized by binding to a specific monoclonal antibody against the amino acid sequence (224–268) that corresponds to the extracellular membrane part of NOX2 (catalytic core of NADPH oxidase), which was released following platelet activation. The enzyme activity is measured spectrophotometrically by the increased absorbance at 450 nm. Values were expressed as pg/mL; intra-assay and inter-assay coefficients of variation were 8.95 and 9.01%, respectively.

**Plasma Detection of ox-LDL**

Ox-LDL levels were measured by commercially available immunoassays (Cusabio, Houston, TX, USA). The optical density of samples was read at 450 nm and was expressed in mU/L. Intra-assay and inter-assay coefficients of variation were <8 and <10%, respectively.

**Plasma and platelets TxB_2_ Assay**

Plasma and platelets Thromboxane (Tx) A_2_ were analyzed by its stable metabolite named TxB_2_ in the supernatant by an ELISA commercial kit (Cusabio, Houston, TX, USA), according to manufacturer instructions. The values were expressed as pg/mL × 10^8^ cells and pg/mL, respectively. Intra- and inter-assay coefficients of variation for TxB_2_ were <8 and <10%, respectively.

**Plasma and platelets H_2_O_2_ production**

The Hydrogen Peroxide (H_2_O_2_) was measured by using a colorimetric assay as previously described [39]. The samples were incubated at room temperature for 20 min, and the reaction product was spectrophotometrically measured at 450 nm and expressed as μM.

**Plasma PCSK9 detection**

Plasma levels of PCSK9 were assessed by a commercial ELISA kit (Abcam, Cambridge, UK). Values were expressed in ng/mL. The intra- and inter-assay coefficients of variation were estimated at 4.4 and 4.6%, respectively.

### 4.1. In Vitro Study

#### 4.1.1. Platelet Preparation

Blood samples added with citrated (3.8%, 1/10 (*v:v*) were taken between 8 and 9 a.m. from healthy subjects (HS, *n* = 5, males 3, females 2, age 37.2 ± 7.0 years) in fasting conditions.

Blood was centrifuged for 15 min at 180 g at room temperature (RT) and the supernatant obtained was separated (2 × 10^5^ platelets/μL) and represents platelet-rich plasma (PRP). Washed platelets (wPLT) were isolated from PRP by consecutive centrifugation steps (10 min at 300× *g* at RT) and resuspended in Tyrode’s buffer (137 mM NaCl, 2.7 mM KCl, 1.0 mM MgCl2, 1.8 mM CaCl2, 20 mM HEPES, 0.35% *w/v* bovine serum albumin (BSA), and 5.6 mM glucose, pH 7.35; Sigma Aldrich, St. Louis MO, USA). Finally, we added prostaglandin E1 (PGE1,1 µM) to prevent platelet activation. Platelet test function was carried out in 3 h.

#### 4.1.2. oxLDL Preparation

Human LDL was obtained from healthy subjects and blood was collected into tubes containing 7.2 mg EDTA. Briefly, blood was centrifuged at 1500 g for 10 min at 4 °C. Next, 250 μL of PBS containing 0.25 mM EDTA were stratified on 750 μL of plasma and tubes were centrifuged at 100,000 rpm for 7 min. The upper 250 μL were removed to eliminate chylomicrons and another 250 μL of PBS with 0.25 mM EDTA were added. Thus, samples were centrifuged at 100,000 rpm for 2.5 h. Next, 250 μL of the upper layer were removed and 150 μL of potassium bromide (KBr) (50%, *w/v*) were added to obtain a density of 1.063 g/mL. Samples were centrifuged at 100,000 rpm for 5 h and the fraction containing LDL was recovered. Lastly, 200 μL of the fraction of LDL was dialyzed with PBS containing EDTA.

Once the LDLs were isolated, they were oxidized by copper sulfate (CuSO_4_). Particularly, LDL (50 µg/mL) was incubated with 10 µL of CuSO_4_ (1.65 µM). Finally, oxLDL concentration was determinate in cell supernatant after oxidation by ELISA kit as reported above.

#### 4.1.3. Platelet Aggregation

wPLTs were resuspended in a Tyrode solution containing 20% of plasma from 5 HeFH patients at center for ox-LDL distribution. Resuspension were obtained using (1) plasma before PCSK9i treatment (ox-LDL median: 24.58 [12.53–17.98–38.04] mU/mL); (2) plasma after PCSK9i treatment (ox-LDL median: 15.99 [11.28–22.21] mU/mL); as negative control was used plasma from 5 healthy subject (ox-LDL median: 8.28 [5.39–8.49] mU/mL).

wPLTs were stimulated with a subthreshold concentration (STC, 0.25 μg/mL), the concentration of agonists was defined as the highest concentration that elicited <20% platelet aggregation of collagen as a primer. STC was used to start aggregation test. To evaluate the role of ox-LDL, before activation, samples were pre-incubated 20 min at 37 °C with or without NOX2ds-tat, that is a selective inhibitor of NOX2 particularly, prevents the translocation of cytosolic p47^phox^ in the membrane and inhibits its interactions with NOX2 [40] (10 μM, AnaSpec) or anti-CD36 (5 μM; Cayman, Ann Arbor, MI, USA) or LOX1 (5 μM; Cayman, Ann Arbor, MI, USA), the receptors on the platelet surface that binds and internalizes ox-LDL or negative control (NC). As negative control of all inhibitors, platelets were incubated with 1% Phosphate-buffered saline (PBS). After incubation, platelets were treated with exogenous ox-LDL (25mU/mL), for 5 min before activation. Platelet aggregation was performed with 2 dual-channel modules Chrono Log Model 700 LT aggregometer (Chrono Log, PA, USA), using siliconized glass cuvettes in constant stirring condition, using Born method [41]. Finally, all samples were centrifuged for 3 min at 3000 rpm and supernatants and pellets were separated and stored at −80 °C for analysis of TXB_2_, and sNOX2-dp as reported below.

### 4.2. Statistical Analysis

All 80 patients completed the study and there was no missing data. Continuous variables were expressed as mean, categorical variables as percentage.

Before–after treatment comparisons were performed using a paired *t*-test. Treatment effect on LDL, ox-LDL, plasma TxB_2_, sNOX2-dp, and plasma PCSK9 were expressed as absolute variation.

Two different stepwise multivariate linear regression analysis models were performed. The first ones were conducted to investigate factors associated with TxB_2_ variation; LDL, ox-LDL, sNOX2-dp, and plasma PCSK9 change were alternatively tested after correction for age, sex, BMI, smoking habits, antiplatelet drugs use, hypertension. The second ones were conducted to test factors associated with ox-LDL change; LDL, sNOX2-dp, and plasma PCSK9 variation were one by one tested after correction for all other variables. “Prior cardiovascular events” variable was not included in the multivariate analyses due to the collinearity with “antiplatelet drugs” variable. Results were considered significant when *p* < 0.05.

All statistical analyses were performed using SPSS 25.0 for Windows (SPSS Institute, Chicago, IL).

## 5. Conclusions

**Figure 4:**
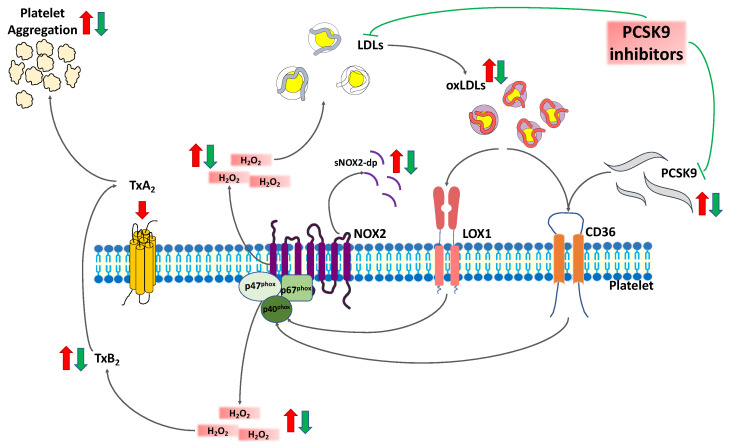
Circulating PCSK9 can bind the CD36 receptor on platelets and activates the downstream signaling including NOX2 activation, ROS production, and platelet activation. PCSK9i modulate platelet function and oxidative stress both reducing NOX2 activation by preventing the interplay between free PCSK9 molecule and CD36 receptor and reducing oxLDL effect on CD36 and LOX1. Green arrow: PCSK9i pathway. Red lines: PCSK9 and ox-LDL pathway. Abbreviation: PCSK9 inhibitors (PCSK9i); Thromboxane A2 (TxA2); soluble NOX2-derived peptide (sNOX2-dp), hydrogen peroxide (H_2_O_2_); low-density lipoprotein (LDL), oxidized-LDL (ox-LDL).

To summarize, we found impaired oxidative stress and PA in patients with HeFH, which decrease after treatment with PCSK9i. Moreover, we demonstrated the key role of NOX2 in this process. Indeed, blocking NOX2 activity by a specific inhibitor, the Nox2ds-tat, a reduction of PA and oxidative stress was observed. Additionally, we obtained the same results blocking CD36, an upstream receptor activating NOX2 cascade. As previously demonstrated, CD36 activity is modulated by both PCSK9 itself and ox-LDL [14,42]. Supporting the hypothesis, exogenous ox-LDL added to after treatment plasma restored the pre-treatment conditions. Instead, the inhibition of both receptors of ox-LDL, CD36, and LOX-1, induced a stronger decrease of PA and oxidative stress confirming their involvement in the process. Putting together these findings, we can conclude that in vivo treatment with PCSK9i modulates platelet function and oxidative stress: (1) reducing NOX2 activation via CD36 by lowering circulating free-PCSK9 and (2) reducing ox-LDL effect on CD36 and LOX1. Thus, the reduction of ox-LDL might be due by both the reduction of LDL oxidation, induced by circulating PCSK9, and the LDL substrate reduction after PCSK9i (Figure 4).

## Figures and Tables

**Table 1:** Patients’ characteristics.

	HeFH Patients Before Treatment (*n* = 80)
Age (years)	57.7 ± 10.8
Female	45%
BMI (kg/m^2^)	26.5 ± 4.2
Smokers (%)	27.5%
Total Cholesterol (mg/dL)	246.0 ± 66.0
HDL cholesterol (mg/dL)	50.8 ± 11.3
LDL cholesterol (mg/dL)	169.2 ± 59.3
Triglycerides (mg/dL)	121.9 ± 53.5
Hypertension (%)	52.5%
Prior Cardiovascular Events (%)	51.5%
Antiplatelet drugs	52.5%
Diabetes	0%
Statins	100%

**Table 2:** Multivariate linear regression analyses.

Δ TxB2					Δ ox-LDL				
	B	E.S	Beta	*p*		B	E.S	Beta	*p*
*Δ PCSK9*	−0.025	0.343	−0.008	0.942	Δ PCSK9	0.153	0.062	0.277	0.016
*Δ ox-LDL*	1.246	0.607	0.233	0.044	-				
*Δ LDL-c*	0.387	0.140	0.316	0.007	Δ LDL-c	0.044	0.027	0.191	0.113
*Δ sNOX2-dp*	0.315	0.595	0.061	0.597	Δ sNOX2-dp	0.223	0.109	0.231	0.045

After adjustment for age, sex, body mass index, antiplatelet drugs, hypertension, smoking habits.

## Data Availability

The data presented in this study are available on request from the corresponding authors.

## Abbreviations

Proprotein convertase subtilisin kexin type 9 (PCSK9); low-density lipoprotein cholesterol receptors (LDLRs); LDL-cholesterol (LDL-c); major adverse cardiovascular events (MACEs); platelet activation (PA); heterozygous familial hypercholesterolemia (HeFH); oxidized-LDL (ox-LDL); Dutch Lipid Clinic Network (DLCN); soluble NOX2-derived peptide (sNOX2-dp); Thromboxane (Tx); hydrogen peroxide (H_2_O_2_); healthy subjects (HS); room temperature (RT); platelet-rich plasma (PRP); washed platelets (wPLT); bovine serum albumin (BSA); prostaglandin E1 (PGE1); negative control (NC). phosphate-buffered saline (PBS).
